# Breast lesion detection through MammoWave device: Empirical detection capability assessment of microwave images’ parameters

**DOI:** 10.1371/journal.pone.0250005

**Published:** 2021-04-13

**Authors:** Lorenzo Sani, Alessandro Vispa, Riccardo Loretoni, Michele Duranti, Navid Ghavami, Daniel Alvarez Sánchez-Bayuela, Stefano Caschera, Martina Paoli, Alessandra Bigotti, Mario Badia, Michele Scorsipa, Giovanni Raspa, Mohammad Ghavami, Gianluigi Tiberi

**Affiliations:** 1 UBT—Umbria Bioengineering Technologies, Perugia, Italy; 2 Breast Screening and Diagnostic Breast Cancer Unit, AUSL Umbria 2, Foligno, Italy; 3 Department of Diagnostic Imaging, Perugia Hospital, Perugia, Italy; 4 School of Engineering, London South Bank University, London, United Kingdom; Seoul National University Hospital, Seoul National University College of Medicine, REPUBLIC OF KOREA

## Abstract

MammoWave is a microwave imaging device for breast lesions detection, which operates using two (azimuthally rotating) antennas without any matching liquid. Images, subsequently obtained by resorting to Huygens Principle, are intensity maps, representing the homogeneity of tissues’ dielectric properties. In this paper, we propose to generate, for each breast, a set of *conductivity weighted* microwave images by using different values of conductivity in the Huygens Principle imaging algorithm. Next, microwave images’ parameters, i.e. *features*, are introduced to quantify the non-homogenous behaviour of the image. We empirically verify on 103 breasts that a selection of these features may allow distinction between breasts with no radiological finding (NF) and breasts with radiological findings (WF), i.e. with lesions which may be benign or malignant. Statistical significance was set at *p*<0.05. We obtained single features Area Under the receiver operating characteristic Curves (AUCs) spanning from 0.65 to 0.69. In addition, an empirical *rule-of-thumb* allowing breast assessment is introduced using a binary score S operating on an appropriate combination of features. Performances of such *rule-of-thumb* are evaluated empirically, obtaining a sensitivity of 74%, which increases to 82% when considering dense breasts only.

## Introduction

Mammography is the gold standard technology for mammographic screening, which has been demonstrated through different randomized controlled trials (RCTs) [1–3] to reduce breast cancer mortality. However, it has some limitations and potential harms, such as the use of ionizing radiation, breast compression and performance restrictions due to the intrinsic nature of x-rays. In particular, breast density is a restrictive property that can prevent breast cancer detection in mammograms of women with radiographically dense breasts [4,5]. In general, women are eligible for biannual screening after the age of 49 in order to minimize the impact of ionizing radiation. Nevertheless, recent studies estimate that breast cancer is diagnosed in 6.6% of women below the age of 40 [4], and an average of 20% of breast cancer cases in Europe occur in women when they are younger than 50 years old [6].

Many efforts are being done to develop non-ionizing technologies which could allow to carry out screening with neither age nor follow-up examination interval restrictions. In this context, microwave imaging appears as a promising technology for breast lesions detection [7]. Microwave imaging methods are developed to discriminate between healthy tissues and tissues with lesions by exploiting their contrast in dielectric properties, i.e. permittivity and conductivity, within the spectrum of microwave frequencies. A high contrast (up to 5) has been reported [7] between healthy breast tissue and malignant tissue, while newer studies confirm a high contrast only between fatty and malignant breast tissues, while it decreases between healthy fibro glandular and malignant tissues [8,9].

Microwave imaging techniques may be classified into two main groups: microwave tomography and ultra-wideband (UWB) radar methods [10]. Microwave tomography is based on inverse scattering algorithms that create maps of permittivity and conductivity; however, inverse scattering approaches could suffer from mathematical instability, which may not converge to a meaningful solution. UWB radar methods instead perform a linear reconstruction of the image, which is a scattering map in arbitrary units.

The exploitation of both microwave imaging techniques has led to the construction of different prototypes, which may differ in hardware and imaging algorithm, i.e., software. Some prototypes are being tested at clinical level: a quite complete review of prototypes at clinical level can be found in [11].

One of these prototypes, Maria system, uses an array of 60 antennas and a matching liquid to carry out radar approach [12] with a sensitivity of 76% [13].

Among prototypes at clinical level, MammoWave requires to operate just two (azimuthally rotating) antennas without any matching liquid, i.e. antennas and breast are in free space. MammoWave has an innovative frequency domain imaging algorithm which is based on Huygens Principle (HP) [14]. This device has been presented, tested and clinically validated [15–17]. Images obtained using the proposed apparatus are intensity maps, given in linear arbitrary units, representing the homogeneity of tissues’ dielectric properties. In this paper, we propose to generate, for each breast, a set of microwave images by using different values of conductivity in the HP imaging algorithm, i.e. *conductivity weighted* microwave images. Next, microwave images’ parameters, i.e. *features*, are calculated to quantify and measure the non-homogenous behaviour of the image. We show that an appropriate selection of image features may allow distinction between breasts with no radiological finding (NF), and breasts with radiological findings (WF), i.e. with lesions which may be benign or malignant. In addition, we show that an appropriate combination and use of image features may allow performance enhancement. The procedure has been empirically verified on 103 breasts, each one with the correspondent output of the radiologist study review obtained using echography and/or mammography and/or MRI.

## Materials and methods

### Microwave apparatus and imaging algorithm

**Fig 1:**
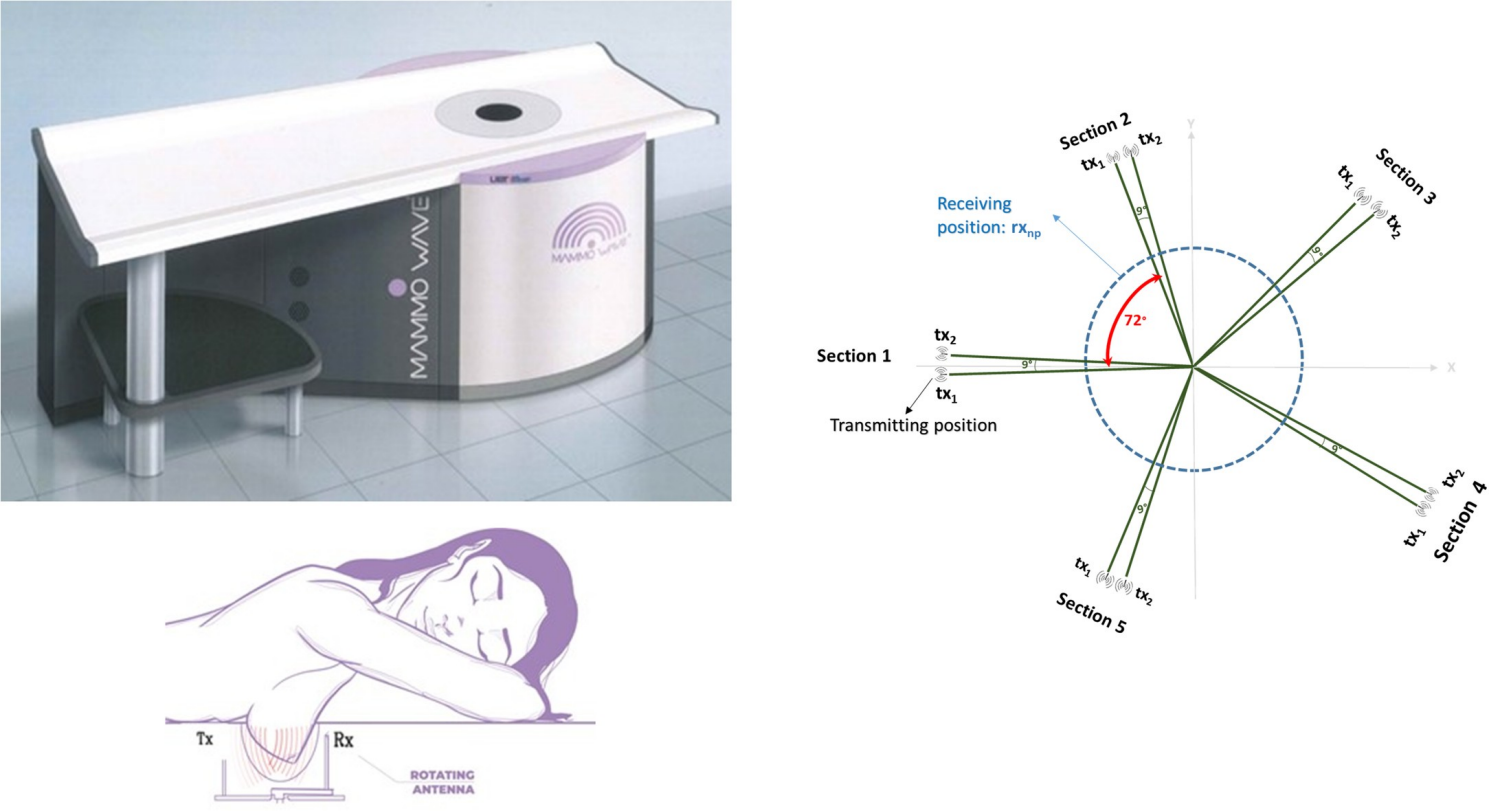
The MammoWave system (top left), consists of an aluminum cylindrical hub containing two antennas, one transmitting and one receiving antenna. The hub is equipped with a hole with a cup, allowing the insertion of the patient’s breast, with the patient lying in a prone position (bottom left). The antennas are installed at the same height, in free space and are able to rotate around the azimuth in order to collect the microwave signals from different angular positions (right).

The MammoWave system, shown in Fig 1 (top left), consists of an aluminum cylindrical hub containing two antennas, one transmitting (tx) and one receiving antenna (rx), which operate in the 1–9 GHz frequency band. The hub is internally covered by microwave absorbers. The hub is equipped with a hole with a cup, allowing the insertion of the patient’s breast, with the patient lying in a prone position. The antennas are installed at the same height, in free space and are able to rotate around the azimuth in order to collect the microwave signals from different angular positions. More details can be found in [15]. The tx and rx are connected to a 2-port VNA (Cobalt C1209, Copper Mountain, Indianapolis, IN) which operates up to 9 GHz. Measurements have been performed recording the complex S21 in a multi-bistatic fashion, i.e. for each transmitting position tx_m_ the receiving antenna is moved to measure the received signal every 4.5°, leading to a total of 80 receiving points rx_np_. Concerning the transmitting positions, all the experiments have been done by employing 10 transmitting position, displaced in 5 sections centered at 0°, 72°, 144°, 216°, and 288°. Fig 1 illustrates the set-up configuration. As Fig 1 (right) shows, in each section the transmitting positions may be displaced by 9°. For each transmitting and receiving position, the complex S21 is collected from 1 to 9 GHz, with 5 MHz sampling.

Assuming that rx can be rotatably moved to measure the received signal at the points ${\mathrm{rx}}_{np}\equiv \left(a_{0},\phi_{np}\right)\equiv {\vec{\rho }}_{np}$ displaced along a circular surface having radius *a*_*0*_, the received signals can be expressed as $S21_{n}^{m,p}\left(a_{0},\phi_{n};{\mathrm{tx}}_{m,p};f\right)$, where *n* = 1,2,…,80, indicates the receiving points; *m* = 1,2…,5 indicates the transmitting sections, *p* = 1,2 and *p’* = 1,2 indicate the position inside each transmitting section; and *f* is the frequency. The received signals are then processed through HP to calculate the field inside the cylinder; such field is then used to generate an image, which is a homogeneity map of dielectric properties. To remove the artefacts [18], here we employ the subtraction between S21 obtained using two measurements belonging to the doublet of the same section. In formula:

$$E_{HP,2D}^{rcstr}\left(\rho ,\phi ;{\mathrm{tx}}_{m,p}-{\mathrm{tx}}_{m,p'};f\right)\propto \overset{N_{FT}}{\underset{n=1}{\sum }}\left(S21_{n}^{m,p}\left(a_{0},\phi_{n};{\mathrm{tx}}_{m,p};f\right)-S21_{n}^{m,p'}\left(a_{0},\phi_{n};{\mathrm{tx}}_{m,p'};f\right)\right)G\left(k_{1}\left|{\vec{\rho }}_{n}-\vec{\rho }\right|\right)$$ (1)

where $(\rho ,\phi )\equiv \vec{\rho }$ is the observation point, *k*_1_ indicates the wave number, and *G* is the Green’s function. The “reconstructed” internal field has been indicated by the string *rcstr* while the string HP indicates that Huygens based procedure will be employed in Eq (1). Note that, if the conductivity of the media is not equal to zero, Eq (1) compensates the attenuation experienced when going into the media.

Assuming we use *N*_*F*_ frequencies *f*_*i*_ in the band B, it follows that the intensity of the image *I* may be obtained through the following equation, i.e. by summing incoherently all the solutions of all the sections:

$$I(\rho ,\phi )=\sum_{m=1}^{5}\sum_{\begin{array}{l} p=1\\ p'=1\\ p\neq p' \end{array}}^{2}\overset{N_{F}}{\underset{i=1}{\sum }}|E_{HP,2D}^{r\mathrm{cst}r}\left(\rho ,\phi ;{\mathrm{tx}}_{m,p}-{\mathrm{tx}}_{m,p'};f_{i}\right){|}^{2}$$ (2)

Image given by Eq (2) is a two-dimensional (2D) image in the azimuthal, i.e. coronal, plane.

### *In-vivo* validation

*In-vivo* validation of MammoWave on volunteers in Perugia Hospital and Foligno Hospital was approved in 2015 by the Ethical Committee of Umbria, Italy (N. 6845/15/AV/DM of 14/10/2015, N. 10352/17/NCAV of 16/03/2017, N 13203/18/NCAV of 17/04/2018). The protocol concerns a feasibility study for detection of breast lesion using the proposed microwave mammogram apparatus, with the aim of quantifying the potential of the proposed microwave mammogram apparatus to be used for medical technology screening. The inclusion criteria allowed female volunteers above 18 years old with intact breast skin and with a radiologist study output obtained through conventional exams (mammography and/or ultrasound and/or magnetic resonance imaging) within the last month. All protocols and procedures were in accordance with both institutional and national ethical standards in research, and with World Medical Association Declaration of Helsinki (1964) and its later amendments or analogous ethical standards. Prior to the trial, all participants have been requested to read and sign both the informative sheet and informed consent form.

We present here the results obtained using a set of data consisting of 103 breasts. Each breast has its own correspondent output of the radiologist study review, which has been used as gold standard for classification of the breasts in two categories: breasts with no radiological finding (NF), and breasts with radiological findings (WF), i.e. with lesions which may be benign or malignant. In this context, radiological study examination included: mammography, performed using Selenia LORAD Mammography System (Hologic, Marlborough, MA), and/or echography, performed using the MyLab 70 xvg Ultrasound Scanner (Esaote, Genova, Italy), and/or magnetic resonance imaging, performed through a 3.0 T MAGNETOM scanner (Siemens Healthcare, Erlangen, Germany). In addition, where possible, the breast type has been classified according to its density, following the scale defined by the American College of Radiology (ACR) which goes from ACR A (almost entirely fatty breasts) to ACR D (extremely dense breasts, which lowers the sensitivity of mammography) [18]. Some details of the detected or suspected lesions have also been collected [19–21]. Moreover, lesions’ final assessment (benign/malignant) has been performed using pathology and/or at least one year of clinical follow-up as reference standards.

Once a subject agrees to participate, she is assisted by the clinical study coordinator; the subject (prone) positions her breast in the cup, which is appropriately integrated in a bed as shown in Fig 1 (bottom left). Specifically, three cups having varying sizes are available, and the clinical study coordinator chose the one that better fits the subject’s breast. Cups are made of polylactic acid (PLA), which has proven to be biocompatible [22]. The thickness of the cup is 1 mm; it has been shown that such thickness does not impact microwave imaging [16].

It is worthwhile pointing out that no matching liquid is used in the apparatus, and no breast compression has to be applied during acquisition.

Microwave images have been first obtained in a cylindrical grid having radius equal to 7 cm (which corresponds to the radius of the receiving antenna), a radial sampling of 1 mm and an azimuthal sampling of 3°. Next, all images have been interpolated on a 2D Cartesian grid having X and Y sampling of 1 mm.

Due to the presence of receiving antenna in free space, the images have been obtained using free space dielectric constant in Eq (1). Instead, concerning the conductivity, for each breast we produced ten different microwave images, i.e. we apply a *conductivity weighing* by varying the conductivity (denoted with σ) from 0 to 0.9 S/m with a sampling of 0.1 S/m when applying Eq (1). We will refer to such microwave images as *conductivity weighted* microwave images (MI), and they will be referred to as MI_*σ*_.

MammoWave acquisition time is approximately 10 minutes (per breast); acquisition is made just once, and then the set of *conductivity weighted* microwave images is produced. Images obtained using the proposed apparatus are intensity maps, given in linear arbitrary units, representing the homogeneity of tissues’ dielectric properties. To allow inter and intra-subject comparison, all images are normalized to unitary average of the intensity.

### Feature extraction

For allowing a quantification of the non-homogenous behaviour of the microwave images, we introduce the following parameters, i.e. *features*:

MIN = Minimum value of the image;
MAX = Maximum value of the image;
MEA = Mean value of the image;
MED = Median value of the image;
VAR = Variance of the image;
MAD0 = Mean absolute deviation of the image;
MAD1 = Median absolute deviation of the image;
KUR = Kurtosis of the image (given by the average value of the two projections in Cartesian grid);
SKE = Skewness of the image (given by the average value of the two projections in Cartesian grid);
M2AVG = (MAX)/(MEA);
ROS1 = (MAX-MIN)/(MEA-MIN);
ROS2 = (MAX-MIN)/(MED-MIN);
ENT = Entropy of the image.

For each *conductivity weighted* image, the previous features are calculated on the full domain of the image, i.e. $\mathrm{feature}\left[{\mathrm{MI}}_{\sigma }^{\mathrm{full}\;\mathrm{image}}\right]$, where they are denoted with the subscript “*_i*”. In addition, for each *conductivity weighted* image, all the features listed above excluding KUR, SKE, ROS1, ROS2 are calculated: on the peak region (a region which is centered in the maximum of the image and it extends to MAX/√2), i.e. $\mathrm{feature}\left[{\mathrm{MI}}_{\sigma }^{\mathrm{peak}}\right]$, where they are denoted with the subscript “*_p*”; and on its complementary, i.e. $\mathrm{feature}\left[{\mathrm{MI}}_{\sigma }^{\mathrm{compl}}\right]$, where they are denoted with the subscript “*_c*”. The ratios between features calculated on the peak region and on its complementary are considered as added features, and they are denoted with the subscript “*_r*”. To summarize, we denote with *feature*[MI_*σ*_] the set of all features of each *conductivity weighted* image.

Next, for each feature, using the gold standard output of the radiological study review (in which breasts have been classified in two categories, NF breasts and WF breasts), we calculate: the mean and standard deviation for the NF breasts, and the mean and standard deviation for the WF breasts.

In addition, for each feature, using the gold standard output of the radiological study review, Welch’s t-test (i.e. a two-sample two-tailed unpooled variances t-test) with α = 0.05 has been performed. Statistical significance was set at *p*<0.05. We also numerically evaluated the receiver operating characteristic (ROC): specifically, for each feature (of each *conductivity weighted* image), we evaluated True Positive (TP) and False Negative (FN) rates. In more details, since TP rate and FN rate depend on the classifier threshold, i.e. the decision offset, we empirically calculated ROC curves by adjusting the decision offset and calculating TP and FN for all possible decision offsets. The area under the curve (AUC) is determined.

### Feature selection and calculations

With the aim of empirically verifying if an appropriate selection and combination of microwave image features may allow discriminating between NF and WF breasts, the following steps are performed for each *conductivity weighted* image:

- for the ROC of each feature, the TP rate obtained for True Negative (TN) rate TN = 0.55, i.e. TP|_TN = 0.55_, is calculated, and the corresponding decision offset is annotated, i.e. *D*_*offset*_{*feature*[MI_*σ*_]};
- we order the feature with decreasing TP|_TN = 0.55_ and we select the first four (after checking that p<0.05 is verified);
- we calculate the average of TP|_TN = 0.55_ on the first four features, i.e. *mean*_*best*5_{*TP*|_*TN* = 0.55_}.

Then, we order the *conductivity weighed* images with decreasing *mean*_*best*5_{*TP*|_*TN* = 0.55_} and we select the first five. In addition, for each breast and for all selected *conductivity weighed* image features, we introduce a binary score S defined as follows:

$$\left\{ \begin{array}{l} \mathrm{if}\;\mathrm{feature}\left[{\mathrm{MI}}_{\sigma }\right]>D_{\mathrm{offset}}\left\{\mathrm{feature}\left[{\mathrm{MI}}_{\sigma }\right]\right\},\;\mathrm{then}\;S=1\\ \mathrm{if}\;\mathrm{feature}\left[{\mathrm{MI}}_{\sigma }\right]\leq D_{\mathrm{offset}}\left\{\mathrm{feature}\left[{\mathrm{MI}}_{\sigma }\right]\right\},\;\mathrm{then}\;S=0 \end{array}\right.$$ (3)

The binary score S is then used for establishing an empirical *rule-of-thumb* allowing assessment of *conductivity weighed* images. Specifically:

if a *conductivity weighed* image has a number of occurrences of S = 1 greater than M, then the *conductivity weighed* image is annotated as *positive*;
if a breast has at least N *positive conductivity weighed* images, such breast is annotated as *positive*.

Performances of the proposed *rule-of-thumb* may be evaluated by empirically calculating the TP rate, i.e. sensitivity, and TN rate, i.e. specificity, by adjusting the decision thresholds M and N. As an example, sensitivity and specificity are empirically calculated here by setting M = 2 and N = 3.

## Results

According to the radiologist study review, a total number of 52 NF (19 dense, i.e. ACR density C and D) and 51 WF (22 dense, i.e. ACR density C and D) breasts were analyzed. The summary of the patient population used in this study is shown in Table 1, while the summary of the radiological study review is given in Table 2. In Table 3, some details of the radiologist study review are given for the 51 WF breasts. Lesions’ final assessment, performed using pathology and/or at least one year of clinical follow-up as reference standards, leads to 30 benign and 17 malignant lesions, while in 4 cases the final assessment is not available.

**Table 1:** Summary of the patient population used in this study.

Total number of patients included in this study	58
Total number of breasts included in this study*	103
Average age of the patients included in this study	52
Number of patients having age 20–49	27
Number of patients having age 50–80	31

*45 out of 58 patients performed MammoWave on both breasts, 13 out of 58 patients performed MammoWave on one breast only.

**Table 2:** Summary of radiological study review for the breasts considered in this study.

	TOT
**NF breasts**	52 (19)
**WF breasts**	51 (22)

In the brackets, the number of dense breasts, i.e. ACR C and ACR D, is given.

**Table 3:** Details of the radiological study review are given for the 51 WF breasts: Age, left (L) or right (R) breast, ACR breast density, mammography/echography BI-RADS, radiologist’s output details such as sizes (mm) and notes (if available), final assessment (Benign/Malignant) obtained using pathology and/or at least one year of clinical follow-up as reference standards (if available).

*Age*	*Breast (L/R)*	*ACR breast density*	*Mammography BI-RADS*	*Echography BI-RADS*	*Radiologist’s output details*: *sizes (mm) and notes (if available)*	*Final assessment (Benign/Malignant)*	*MammoWave rule-of-thumb output*
48	L	D	3	-	Microcalcifications	Benign	Positive
65	L	C	4	-	Cluster of microcalcifications	Benign	Positive
40	L	B	2	2	Three masses: 15 mm, 21 mm and 23 mm	Benign	Positive
	R	B	2	2	Microcalcifications	Not available	Positive
52	L	C	5	-	Microcalcifications	Malignant	Positive
47	L	D	2	2	Microcalcifications	Benign	Negative
55	R	C	2	2	1.6 mm microcalcifications	Benign	Positive
	L	C	2	2	3.8 mm microcalcifications	Benign	Negative
51	L	C	2	2	Presence of metallic marker	Benign	Positive
54	R	A	2	2	Microcalcifications	Benign	Positive
77	R	D	-	5	17 mm mass	Malignant	Positive
61	R	C	4	-	Multifocal lobular type suspected carcinoma (MRI BI-RADS 4)	Malignant	Positive
	L	C	2	-	Macrocalcification and Focal contrast enh. (MRI BI-RADS 3)	Not available	Positive
50	L	B	2	2	10 mm mass	Benign	Positive
67	L	C	4	-	Microcalcifications	Malignant	Negative
49	L	A	3	-	Microcalcifications	Benign	Positive
70	L	D	3	4	Mass	Malignant	Positive
42	L	C	2	3	7 mm mass, hypoechoic	Benign	Negative
67	L	B	3	-	Architectural distortion	Benign	Positive
56	R	B	4	4	31 mm mass, hypoechoic, irregular borders	Malignant	Positive
43	R	D	1	3	12 mm mass	Benign	Positive
51	L	C	3	-	Microcalcifications	Benign	Positive
59	L	B	-	4	11 mm areolar, suspicious of malignancy	Malignant	Positive
40	L	D	2	2	30 mm mass	Benign	Positive
35	R	C	2	3	7 mm, hypoechoic	Benign	Positive
37	L	A	2	3	25 mm mass	Benign	Negative
43	R	B	3	2	Microcalcifications	Malignant	Negative
54	R	B	2	2	18 mm mass	Benign	Negative
49	L	A	2	3	16 mm mass	Benign	Positive
56	L	D	4	4	27 mm mass	Malignant	Positive
63	L	A	3	4	6 mm mass	Malignant	Positive
55	R	C	4	4	23 mm mass	Malignant	Positive
	L	C	2	2	Multiple cysts	Benign	Positive
64	R	B	3	-	1.6 mm microcalcifications	Benign	Negative
37	R	-	-	3	15.4 mm mass	Benign	Positive
	L	-	-	2	Multiple cysts	Not available	Positive
76	R	-	-	3	13 mm mass	Malignant	Negative
45	R	B	4	4	14 mm mass	Malignant	Positive
72	L	B	4	4	22 mm mass	Malignant	Positive
57	L	-	-	4	14 mm mass	Malignant	Negative
20	L	-	-	2	16 mm mass	Benign	Negative
46	R	B	2	2	12 mm mass	Benign	Positive
78	L	A	-	4	18 mm mass, hypoechoic	Malignant	Positive
	R	A	3	2	Microcalcifications	Not available	Positive
62	R	B	4	-	Opacity	Malignant	Negative
44	L	B	3	3	24 mm mass	Benign	Positive
57	R	A	3	-	Opacity	Benign	Positive
63	R	A	3	-	Opacity	Benign	Negative
40	L	D	1	2	33 mm mass	Benign	Positive
	R	D	1	2	Two masses: 7 mm and 22 mm, hypoechoic	Benign	Positive
46	L	B	2	2	12 mm mass	Benign	Positive

In the last column, the MammoWave *rule-of-thumb* output is given.

**Fig 2:**
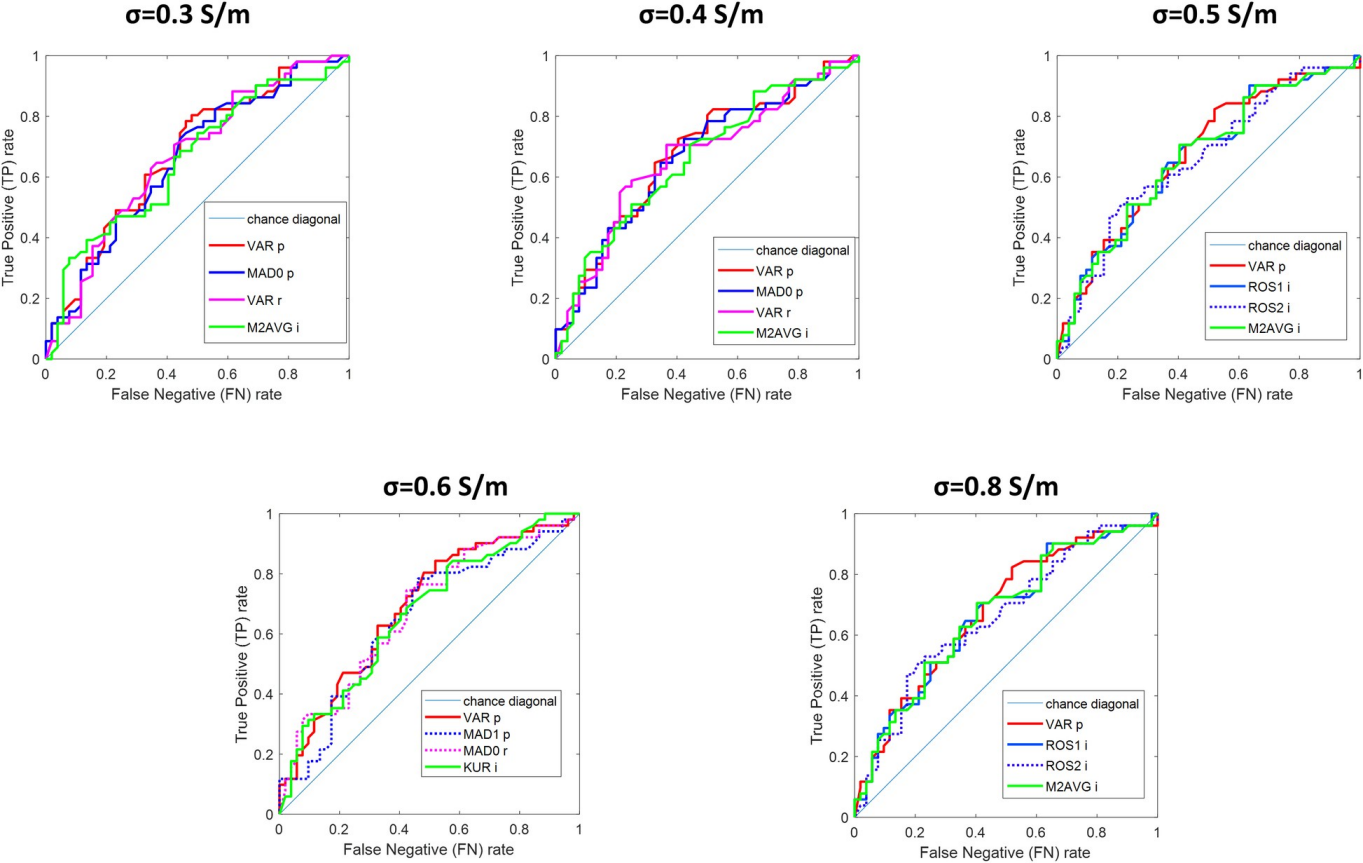
ROC curves of the selected features for the selected *conductivity weighed* images; the value of the correspondent conductivity (expressed in S/m) is given above each figure.

The selected features for the selected *conductivity weighed* images are listed in Table 4. For each feature, we indicate: the mean and standard deviation for the NF breasts; the mean and standard deviation for the WF breasts; the decision offset corresponding to TN = 0.55; Welch’s t-test score and p-value; the AUC. ROC curves of the selected features are shown in Fig 2.

**Table 4:** List of the selected features for the selected *conductivity weighed* images.

	Mean(NF)	Std(NF)	Mean(WF)	Std(WF)	D_offset_	t-test	p-value	AUC
**σ = 0.3 S/m**								
’VAR_p’	0.152	0.070	0.201	0.085	0.144	1	0.0019	0.68
’MAD0_p’	0.308	0.073	0.353	0.077	0.305	1	0.0056	0.66
’VAR_r’	1.115	0.604	1.397	0.612	1.060	1	0.0032	0.67
’M2AVG_i’	2.143	0.320	2.317	0.350	2.124	1	0.0060	0.66
**σ = 0.4 S/m**								
’VAR_p’	0.216	0.081	0.275	0.107	0.201	1	0.0017	0.68
’MAD0_p’	0.370	0.070	0.416	0.084	0.362	1	0.0037	0.66
’VAR_r’	1.368	0.631	1.698	0.742	1.267	1	0.0035	0.66
’M2AVG_i’	2.267	0.346	2.478	0.392	2.236	1	0.0049	0.66
**σ = 0.5 S/m**								
’VAR_p’	0.277	0.094	0.349	0.125	0.254	1	0.0008	0.69
’MAD1_p’	0.358	0.076	0.401	0.090	0.339	1	0.0089	0.65
’MAD0_r’	1.208	0.233	1.364	0.288	1.170	1	0.0024	0.67
’KUR_i’	2.793	0.641	3.162	0.793	2.632	1	0.0034	0.67
**σ = 0.6 S/m**								
’VAR_p’	0.335	0.108	0.416	0.147	0.311	1	0.0016	0.68
’M2AVG_i’	2.499	0.386	2.753	0.447	2.479	1	0.0035	0.68
’MAX_p’	2.499	0.386	2.753	0.447	2.479	1	0.0035	0.66
’ROS1_i’	2.548	0.392	2.805	0.453	2.530	1	0.0036	0.666
**σ = 0.8 S/m**								
’M2AVG_i’	2.700	0.420	2.981	0.491	2.654	1	0.0025	0.67
’MAX_p’	2.700	0.420	2.981	0.491	2.654	1	0.0025	0.67
’ROS1_i’	2.731	0.423	3.013	0.494	2.674	1	0.0027	0.67
’ROS2_i’	2.908	0.708	3.346	0.857	2.773	1	0.0040	0.66

For each feature, we indicate: The mean and standard deviation for the NF breasts; the mean and standard deviation for the WF breasts; the decision offset corresponding to TN = 0.55; Welch’s t-test score and p-value; the AUC.

**Fig 3:**
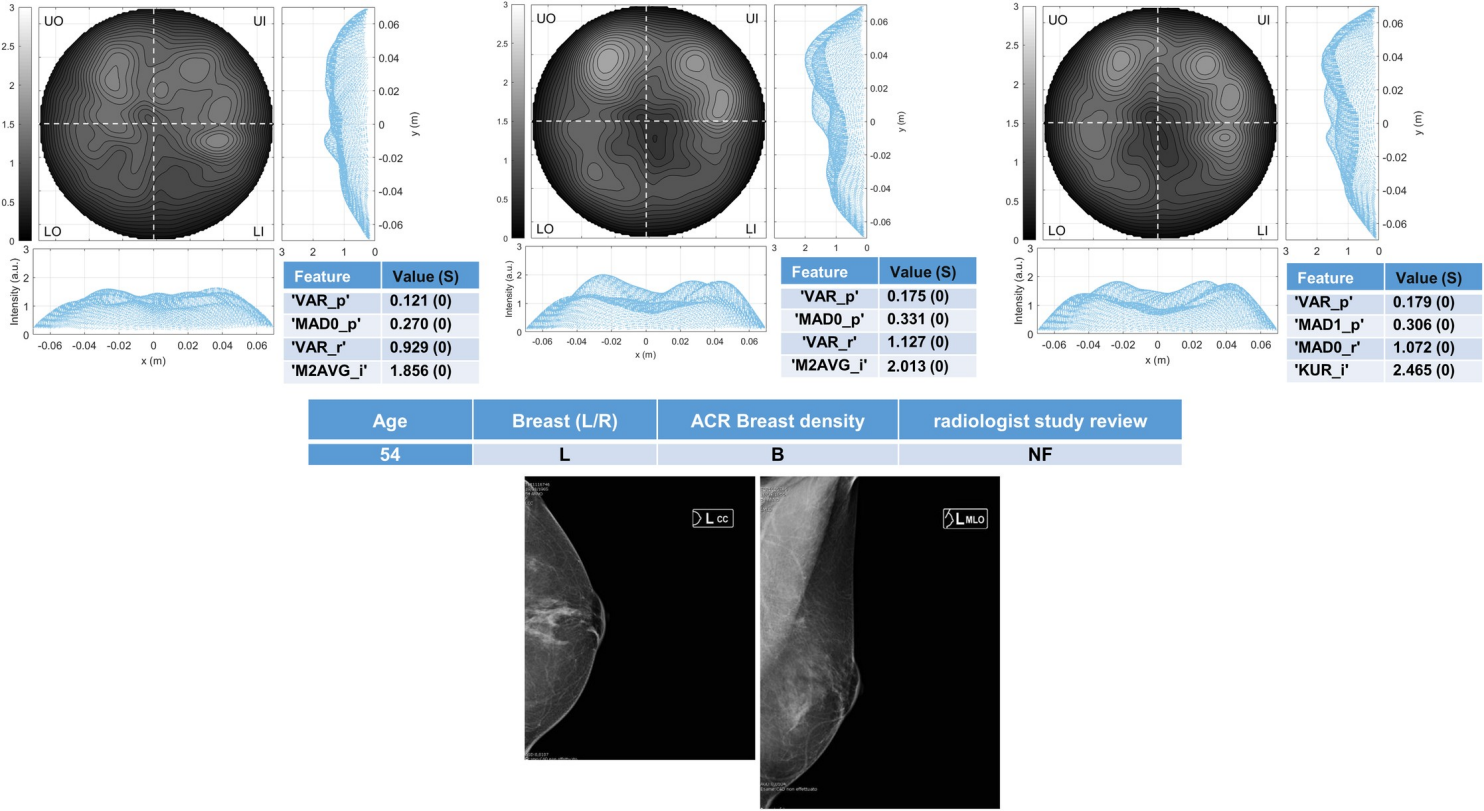
The radiologist study review “NF” for this scattered area of fibroglandular density (ACR B) breast has been obtained with the support of mammography images given in the bottom row. Microwave images, normalized to unitary average of the intensity, are given in the top row for three different conductivity weighting (from left to right: 0.3 S/m, 0.4 S/m and 0.5 S/m, respectively). X and Y are given in meters; intensity is in arbitrary units. All microwave images show a quite homogeneous behavior. The binary score S (given in the inserted tables) is 0 for the all the features. The proposed *rule-of-thumb* classifies this breast as negative.

**Fig 4:**
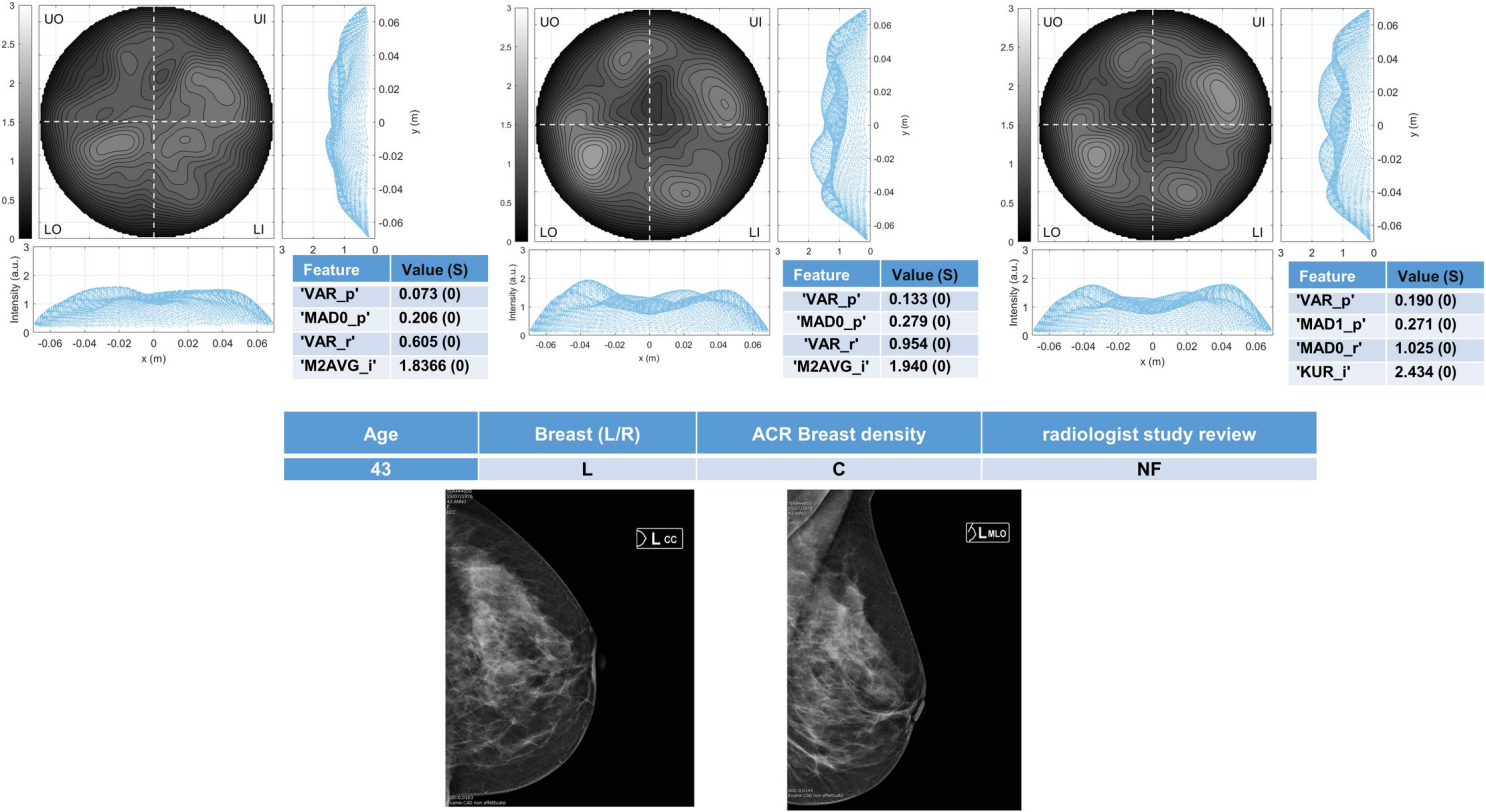
The radiologist study review “NF” for this heterogeneously dense (ACR C) breast has been obtained with the support of mammography images given in the bottom row. Microwave images, normalized to unitary average of the intensity, are given in the top row for three different conductivity weighting (from left to right: 0.3 S/m, 0.4 S/m and 0.5 S/m, respectively). X and Y are given in meters; intensity is in arbitrary units. All microwave images show a quite homogeneous behavior. The binary score S (given in the inserted tables) is 0 for the all the features. The proposed *rule-of-thumb* classifies this breast as negative.

**Fig 5:**
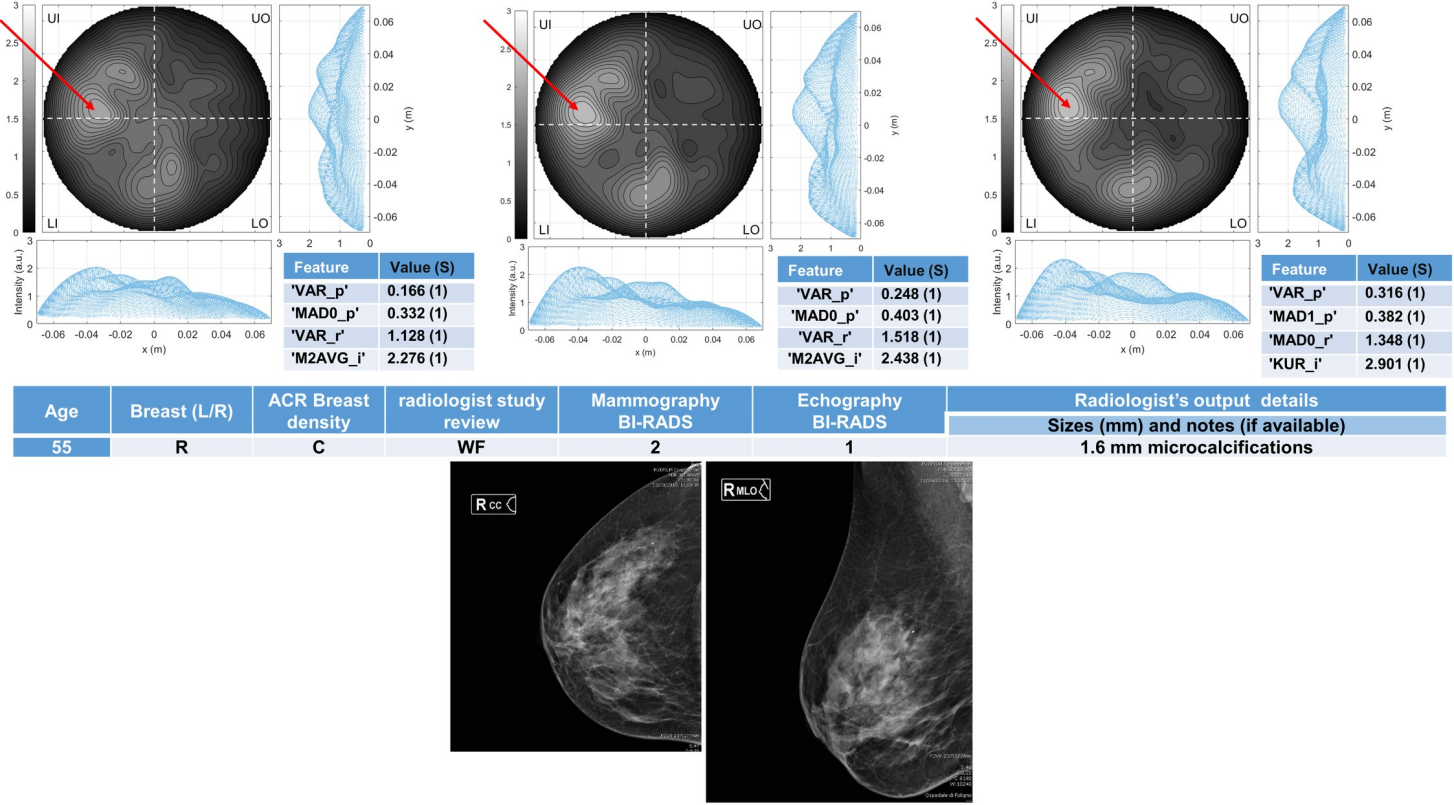
The radiologist study review “WF” for this heterogeneously dense (ACR C) breast has been obtained with the support of mammography images given in the bottom row, giving as output the presence of microcalcifications of 1.6 mm. The Echography BI-RADS is 1 and the Mammography BI-RADS is 2. The final assessment is benign lesion. Microwave images, normalized to unitary average of the intensity, are given in the top row for three different conductivity weighting (from left to right: 0.3 S/m, 0.4 S/m and 0.5 S/m, respectively). X and Y are given in meters; intensity is in arbitrary units. All microwave images show a non-homogeneous behavior, with a main peak indicated by the red arrows. The binary score S (given in the inserted tables) is 1 for the all the features. The proposed *rule-of-thumb* classifies this breast as positive.

**Fig 6:**
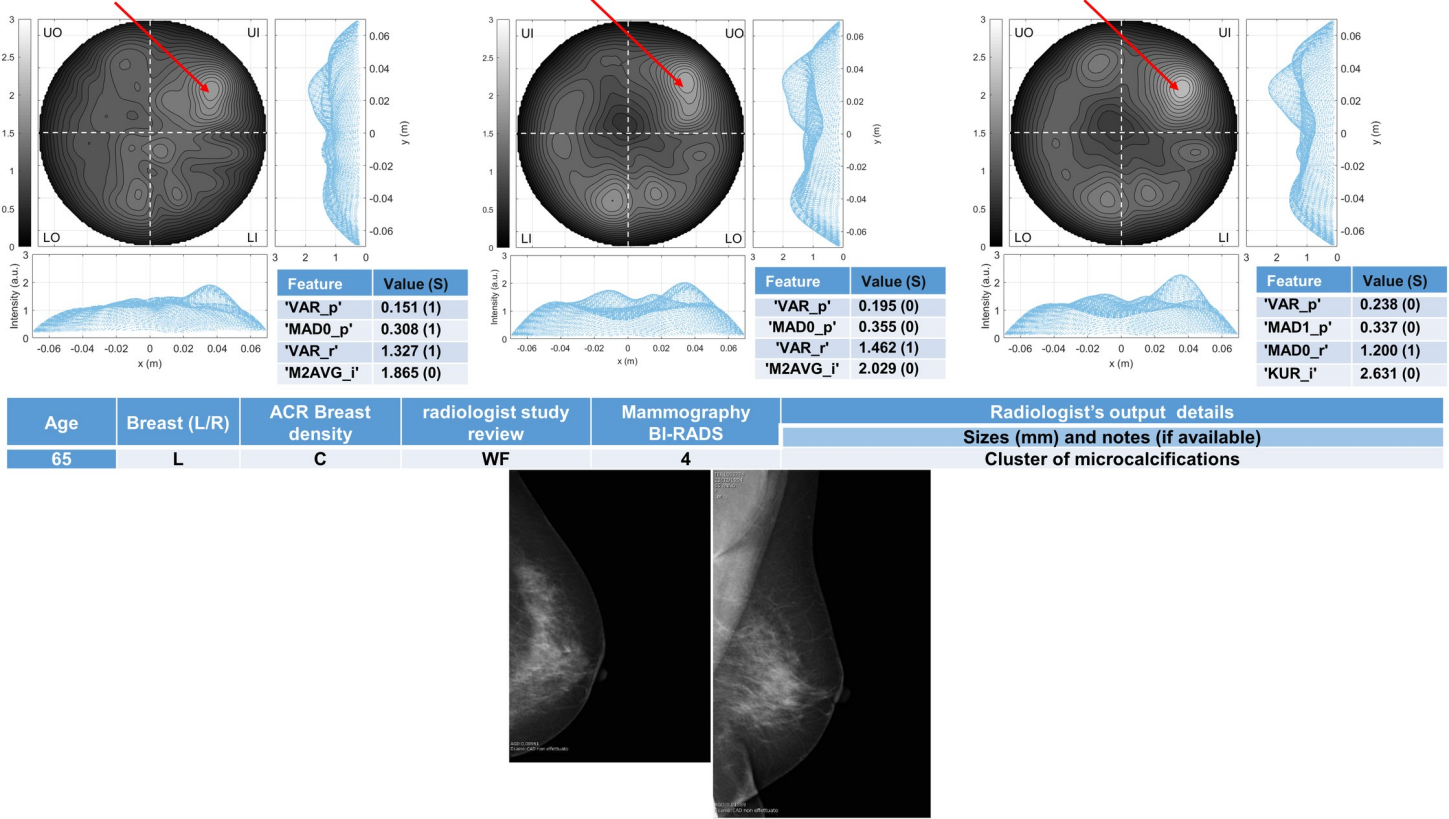
The radiologist study review “WF” for this heterogeneously dense (ACR C) breast has been obtained with the support of mammography images given in the bottom row, giving as output the presence of a cluster of microcalcifications. The Mammography is BI-RADS 4. The final assessment is benign lesion. Microwave images, normalized to unitary average of the intensity, are given in the top row for three different conductivity weighting (from left to right: 0.3 S/m, 0.4 S/m and 0.5 S/m, respectively). X and Y are given in meters; intensity is in arbitrary units. All microwave images show a non-homogeneous behavior, with a main peak indicated by the red arrows. From the binary score S (given in the inserted tables) we note that the number of occurrences of “1” is three when σ = 0.3 S/m, one when σ = 0.4 S/m and one when σ = 0.5 S/m. The proposed *rule-of-thumb* classifies this breast as positive.

**Fig 7:**
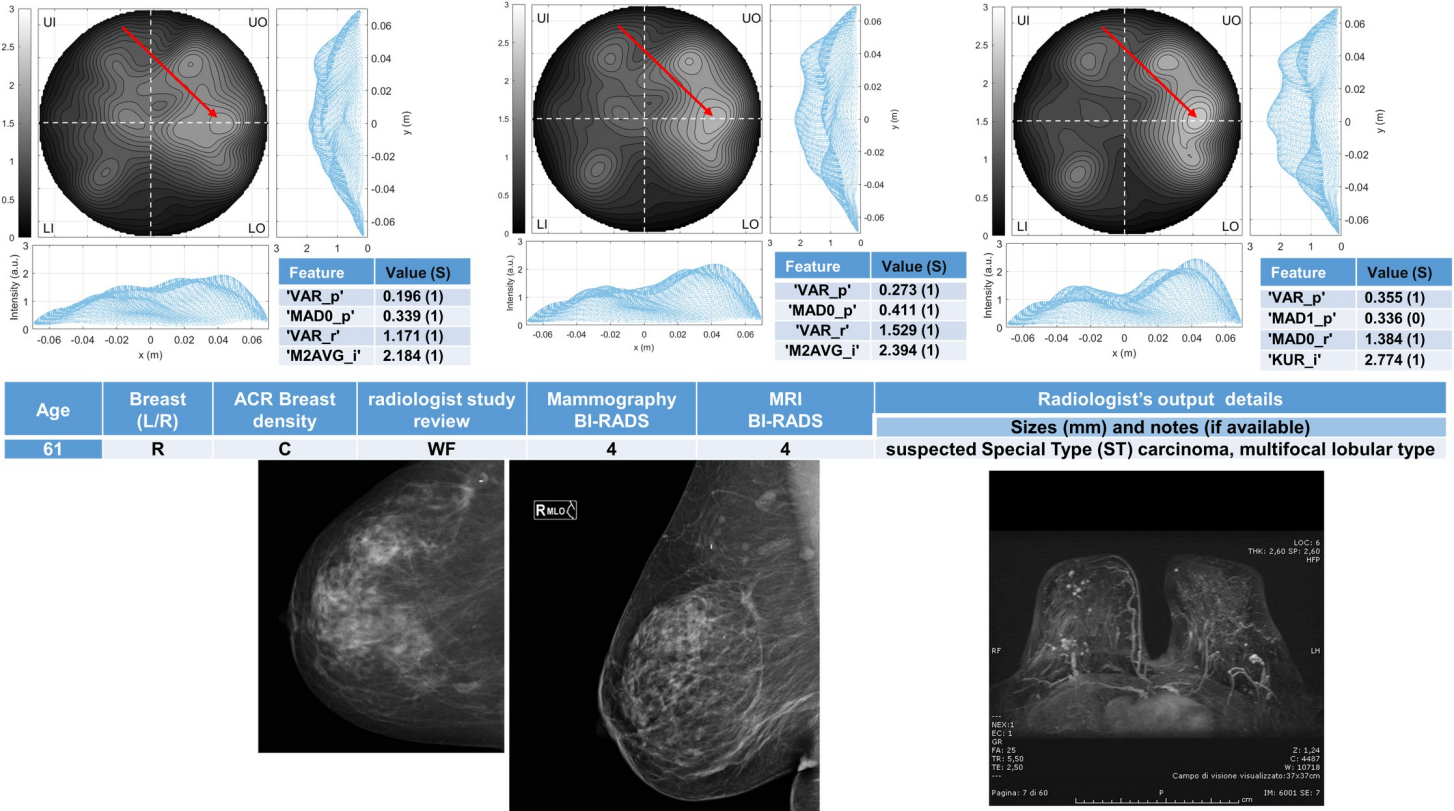
The radiologist study review “WF” for this heterogeneously dense (ACR C) breast has been obtained with the support of mammography and MRI images given in the bottom row, giving as output the presence of a suspected Special Type (ST) carcinoma, multifocal lobular type. The Mammography BI-RADS is 4 and the Magnetic Resonance Imaging (MRI) BI-RADS is 4. The final assessment is malignant lesion. Microwave images, normalized to unitary average of the intensity, are given in the top row for three different conductivity weighting (from left to right: 0.3 S/m, 0.4 S/m and 0.5 S/m, respectively). X and Y are given in meters; intensity is in arbitrary units. All microwave images show a non-homogeneous behavior, with a main peak indicated by the red arrows. From the binary score S (given in the inserted tables) we note that the number of occurrences of “1” is four when σ = 0.3 S/m, four when σ = 0.4 S/m and three when σ = 0.5 S/m. The proposed *rule-of-thumb* classifies this breast as positive.

**Fig 8:**
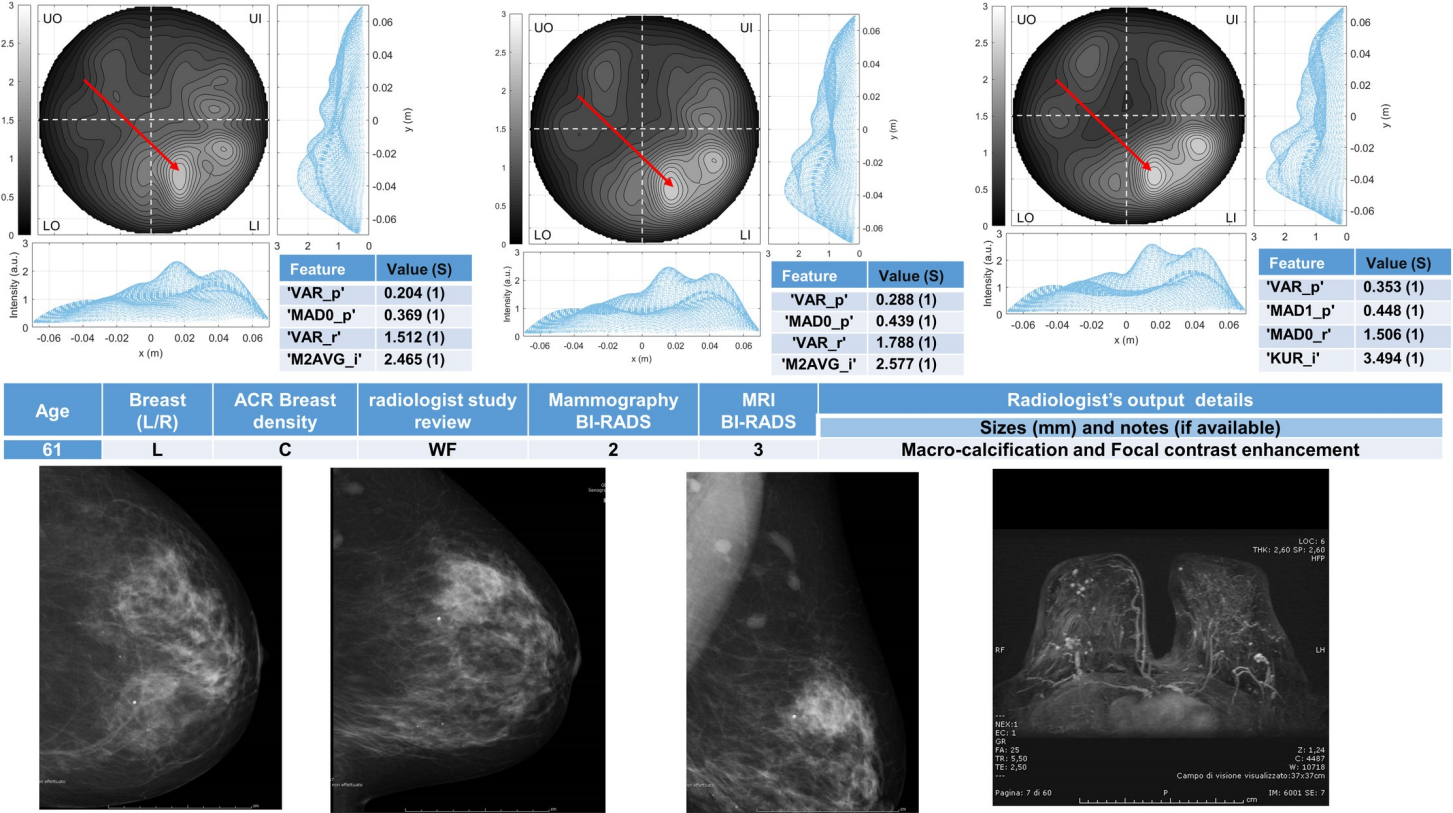
The radiologist study review “WF” for this heterogeneously dense (ACR C) breast has been obtained with the support of mammography and MRI images given in the bottom row, giving as output the presence of a macro-calcification and a focal contrast enhancement. The Mammography BI-RADS is 2 and the Magnetic Resonance Imaging (MRI) BI-RADS is 3. The final assessment is not available. Microwave images, normalized to unitary average of the intensity, are given in the top row for three different conductivity weighting (from left to right: 0.3 S/m, 0.4 S/m and 0.5 S/m, respectively). X and Y are given in meters; intensity is in arbitrary units. All microwave images show a non-homogeneous behavior, with a main peak indicated by the red arrows; a slightly lower peak in the same quadrant may be also noted. The binary score S (given in the inserted tables) is 1 for the all the features. The proposed *rule-of-thumb* classifies this breast as positive.

Six breasts are shown here in more details as six test cases, each one with three of the selected *conductivity weighed* microwave images (obtained for conductivities equal to 0.3 S/m, 0.4 S/m and 0.5 S/m, respectively). Figs 3 and 4 refer to NF breasts, while Figs 5–8 refer to WF breasts. Microwave images, normalized to unitary average of the intensity, are given here as 2D images in the azimuthal, i.e. coronal, plane; the images are divided into four quadrants corresponding to breast Upper-Outer (UO) quadrant; Upper-Inner (UI) quadrant; Lower-Outer (LO) quadrant; Lower-Inner (LI) quadrant. Moreover, 1D intensity projection on X and Y is displayed in the inserts. X and Y are given in meters; intensity is in arbitrary units. In each figure, the tables given as inserts of microwave images show the values of the correspondent selected features; in the same tables, for each feature we also report the binary score S in brackets, calculated from Eq (3).

For each one of the six test cases, the output and main findings of the radiologist study review, with the correspondent conventional images, is also given. BI-RADS categories are also given for WF breasts. In more details, Figs 5 and 6 refer to breasts with microcalcifications and for both cases the final assessment is benign lesion; Fig 7 refers to breast with suspected carcinoma and the final assessment is malignant lesion; Fig 8 refers to breast with a macro-calcification and focal contrast enhancement (the final assessment is not available).

Performances of the *rule-of-thumb* introduced above are evaluated empirically, after setting M = 2 and N = 3. We obtain a sensitivity of 38/51 ~ 74% (which increases to 18/22 ~ 82% when considering dense breasts only, i.e. ACR C and ACR D), with a specificity of 32/52 ~ 62%. Sensitivity performances of the *rule-of-thumb* are summarized in Table 5, while the performance details for each one of the 51 WF breasts can be found in the last column of Table 3. In Table 5, MammoWave *rule-of-thumb* sensitivity is given also for benign and malignant findings, separately; specifically, for benign findings we obtain a sensitivity of 21/30 ~ 70% (which increases to 11/14 ~ 78% when considering dense breasts only) while for malignant findings we obtain a sensitivity of 12/17 ~ 71% (which increases to 6/7 ~ 85% when considering dense breasts only).

**Table 5:** MammoWave *rule-of-thumb* sensitivity is summarized (second row) for the WF breasts (both full set and dense breasts only): Sensitivity is expressed as numerator/denominator (where the numerator represents the number of *rule-of-thumb* positive identification and the denominator represents the total number of WF breasts) and in percentages (given in brackets and rounded to nearest whole number).

	MammoWave *rule-of-thumb* sensitivity	MammoWave *rule-of-thumb* sensitivity: dense breasts only
all WF breasts	38/51 (74%)	18/22 (82%)
benign finding	21/30 (70%)	11/14 (78%)
malignant finding	12/17 (71%)	6/7 (85%)

Similarly, MammoWave *rule-of-thumb* sensitivity is summarized for benign (third row) and malignant (fourth row) findings, separately (both full set and dense breasts only).

## Discussion and conclusion

Microwave images obtained using the proposed apparatus are intensity maps, given in linear arbitrary units, representing the homogeneity of breast’s dielectric properties. MammoWave does not use any patient-specific estimation, which means that breast images are generated without any prior knowledge of patient-specific breast dielectric properties. In more details, the images have been obtained using free space dielectric constant in Eq (1). Concerning the conductivity, for each breast we produced ten different microwave images by varying the conductivity from 0 to 0.9 S/m (in agreement with the breast conductivity average values reported in [10]).

From visual inspection of microwave images, it can be pointed out that microwave images of WF breasts have a more non-homogenous behaviour with respect to NF breast. This confirms what was previously highlighted in [15,16], also through the use of phantom measurements, i.e. the contrast in dielectric properties between breast lesions and the surrounding tissues generates a peak in microwave images. Interestingly, small microcalcifications (1.6 mm) also lead to non-homogenous behaviour which can be visually appreciated.

With the aim of discriminating between WF and NF breasts, some dedicated features have been introduced and selected. Such features allow a quantification of the non-homogeneity of the microwave images: some of them describe the entire image [15,16], while others describe the peak region [23]. From Table 4, it is possible to verify that p-values of all the selected features are <0.001; thus, it follows that selected features are statistically robust in discriminating between WF and NF breasts. False Discovery Rate (FDR) correction for multiple comparisons may also be applied to the statistical tests: we verified that this leads to a slight increase of the p-values for the selected features, which remain statistically robust in discriminating between WF and NF breasts. Yet, from Table 4 it is clear that overlap exists among WF and NF breasts features. AUC of selected features span from 0.65 to 0.69. In addition, we also calculated AUCs of the selected features when considering dense breasts only, noting an increase up to 0.77.

The binary score S operating on the combination of features may be used for establishing an empirical *rule-of-thumb* allowing breast assessment; the underlying idea is that a “large number of occurrences of 1” may indicate a WF breast, while a “large number of occurrences of 0” may indicate a NF breast. From the examples given here, it can be noted that microcalcifications in an ACR C breast may have a “large number of occurrences of 1” in microwave images with lower conductivity weighting. Conversely, a carcinoma in an ACR C breast have a “large number of occurrences of 1” also in microwave images with higher conductivity weighting. Indeed, also from visual inspection it can be seen that a carcinoma in an ACR C may be better highlighted in microwave images with higher conductivity weighting. It follows that the use of a range of conductivity weighting when generating microwave images may be beneficial in detecting different kinds of lesions.

Performances of the proposed *rule-of-thumb* have been evaluated by empirically calculating the sensitivity (after setting M = 2 and N = 3), obtaining an overall value of 74%, with a specificity of 62%. Sensitivity increases to an overall value of 82% when considering dense breasts only. From the results obtained when considering benign and malignant findings, separately, it appears that MammoWave sensitivity is similar for both benign and malignant lesions, i.e. 70% and 71%, respectively (it should be noted that such values are lower than the overall value, since in 4 cases the final assessment is not available). Higher breast density has a positive impact in detection, increasing MammoWave sensitivity for both benign and malignant lesions to 78% and 85%, respectively. These values are in agreement with [12,13], where symptomatic patients only have been recruited; specifically, it is reported that sensitivity is 74% and 76% for benign and malignant lesions, respectively, and it increases to 79% (in both benign and malignant lesions) when considering dense breasts [13].

A patient-specific knowledge of dielectric properties may lead to a further improvement in sensitivity/specificity [23,24], By comparing the performances of the proposed *rule-of-thumb* (which combines many features) with respect to single features ROC curves (given in Fig 2), we can appreciate an increase in sensitivity; this is in agreement with [25], where a multi-feature analysis of Magnetic Resonance breast images has been performed.

A limitation of this investigation is that we did not consider pre-menstrual information of the subjects, due to such information not being available. A further limitation of this investigation is that, concerning *rule-of-thumb* modality for breast assessment, the impact on detection capabilities of the features/methods selection procedure, number of selected features/methods as well as of features’ correlation has not been investigated. Specifically, the number of selected features, i.e. 4, and methods, i.e. 5, has been selected arbitrarily. Moreover, ROC curves have been empirically calculated. However, it should be emphasized that the main aims of this paper are: i) to verify if a selection of *features* obtained from a range of *conductivity weighted* microwave images may allow discriminating between NF and WF breasts; ii) to verify if an appropriate combination and use of microwave image features may achieve performance enhancement versus single feature. Finally, it should be pointed out that, for this study, each breast has its own correspondent output of the radiologist study review, which has been used here as gold standard for classification of the breast into two categories: NF and WF breast. Some details of the detected or suspected lesions (such as BI-RADS categories, sizes and notes) have been collected throughout the study and, thus, they are shown here, but they are not used in statistical analysis.

Further work on MammoWave, which has recently received CE Mark (Conformité Européenne) approval, is ongoing and more clinical trials are planned with the aim of improving clinical evidence on the use of microwave imaging in the breast screening pathway. In addition, while our main current goal is discriminating between NF and WF breasts, dedicated clinical trials are also planned for quantifying capability in distinguishing malignant lesions.
